# State of the art in post-mortem forensic imaging in China

**DOI:** 10.1080/20961790.2017.1337501

**Published:** 2017-06-19

**Authors:** Yijiu Chen

**Affiliations:** Institute of Forensic Science, Ministry of Justice, PRC, Shanghai, China

**Keywords:** Forensic science, post-mortem forensic imaging, post-mortem computer tomography, post-mortem magnetic resonance imaging, post-mortem computer tomography angiography, finite element analysis

## Abstract

The autopsy and histopathologic examination are traditional and classic approaches in forensic pathology. In recent years, with the tremendous progresses of computer technology and medical imaging technology, the developed post-mortem computer tomography, post-mortem magnetic resonance imaging and other new methods provide non-invasive, intuitive, high-precision examining methods and research tools for the forensic pathology. As a result, the reconstruction of the injury as well as the analysis of injury mechanism has been essentially achieved. Such methods have become popular in the research field of forensic science and related work has also been carried out in China. This paper reviews the development and application of abovementioned post-mortem forensic imaging methods in China based on the relevant literature.

## Introduction

In the context of globalization, informatization, and the democratization and legalization of society, the importance of scientific evidence has been increasingly highlighted, and the conclusions made by forensic experts are important scientific evidence. Traditional forensic medical examination mainly relies on the visual observation and experience-based judgment in the processes of autopsy and histopathological examination. The impartiality and scientific integrity of the forensic evidences has been repeatedly questioned, and the faulty forensic evidence often causes the contradictions and conflicts between people involved in the case and investigators.

In recent years, with the development of science and technology, the integration of forensic pathology, medical imaging technology, computer technology, telematics, information processing technology, biomechanics and other disciplines formed a series of new post-mortem forensic imaging methods, bringing a revolutionary breakthrough in forensic science [1,2]. Compared to traditional technologies, post-mortem forensic imaging methods are non-invasive, repeatable, intuitive, etc., and are more conducive to resolving the key issues in cases [3,4]. This paper introduces the currently well-developed post-mortem forensic imaging methods including post-mortem computer tomography (PMCT), post-mortem magnetic resonance imaging (PMMR), post-mortem computer tomography angiography (PMCTA) and finite element analysis (FEA) of human injury biomechanics based on post-mortem forensic imaging data, and also elaborate the state of the art in those methods in China.

## Post-mortem forensic imaging based on modern imaging technology

In all cases involving the death during the litigation process, to identify the cause of death is the basis for the case handling. The primary tasks are to determine the cause of death, manner of death, and assessment of fatal and non-fatal injuries, and scene reconstruction should be performed when it is possible. Forensic autopsy is recognized as the gold standard for a clear cause of death. Because of the limitations in the examinations of special parts of the body, the change or destruction of the body due to the autopsy operation and various internal and external factors, the limitations in the technologies for histopathological examination, etc., the autopsy cannot always provide sufficient information to determine the cause of death [5]. Furthermore, a traditional autopsy is a destructive examination, and there have been resistance and objection by the family of the deceased for centuries, especially due to the repulsion and rejection by some religious and cultural traditions [6]. The development of multi-detector computed tomography (MDCT), magnetic resonance imaging (MRI) and other medical imaging technologies provides a non-invasive/minimally invasive autopsy approach for the forensic examination, i.e. post-mortem forensic imaging. This technology can provide a clear observation of lesions and damages on cadaveric organs, bones and other structures [4,7]. The image data of various parts of the bodies can be saved, favouring the future re-examination and consultation, and this technology has a significant value for the detection of forensic injury and determination of the cause of death [2,8].

Post-mortem forensic imaging refers to the use of modern medical imaging and computer technologies to obtain the images of internal body structures and organs of human body in a non-invasive or minimally invasive manner, detect the injuries, diseases and other morphological changes in human body, and provide the evidence to determine the cause and manner of death [9,10]. Post-mortem forensic imaging includes MDCT and MRI, microscopic radiation scanning technology, and volume rendering scanning/voxel scanning and imaging technology [1]. With these technologies, the valuable information inside the body can be collected and saved as data. Two-dimensional (2D) or three-dimensional (3D) images of the exterior and interior injuries and lesions in the body are generated after computer processing, making it easy for observation, discussion and remote consultation [2,11]. After the post-mortem forensic imaging follows the traditional autopsy, the findings of the two methods can be compared and analysed, which will yield a more objective judgment compared with that obtained by a classical autopsy alone [5,12]. Currently, post-mortem forensic imaging methods has been widely used in forensic practice, crime scene reconstruction, injury detection and determination of the cause of death, and it has a high practicability and shows sustainable development and progress.

Post-mortem angiography, including PMCTA and post-mortem magnetic resonance angiography (PMMRA), is a means for the diagnosis of vascular disease and injury by injecting the contrast agent into the circulation system [13–15]. The vessels filled with contrast agents are examined by scanning of the corpse. The present contrast agents include suspensions, oily liquid, water-soluble agents, mould materials, mixtures and so on [16–18], and the injection methods include single-organ angiography [19,20] and whole-body angiography [18,21–24]. Studies have shown that PMCTA/PMMRA can clearly show the vascular malformations, and lesions in the body, and the shape, size and location of vascular rupture, and can be used to guide the anatomic path and assist the determination of the cause of death [25,26].

Systemic researches of post-mortem forensic imaging emerged in Switzerland in the early twenty-first century [1,2,4]. Then the technology has attracted significant attention in the global forensic community and been rapidly developed. Enormous research with great achievements has been made in the field of forensic science. Currently, Switzerland [27–40], the United States [41–43], Japan [44–46], Britain [47–50] and many other countries have carried out post-mortem forensic imaging projects that integrate multiple technologies, including 3D optical scanning, PMCT, PMMR, PMCTA, PMMRA, etc. Those projects focused on the determination of the cause of death, forensic pathological detection, vital reactions, the reconstruction, reproduction and imaging of injuries, and other key aspects in forensic science.

### Post-mortem forensic imaging in China

**Figure 1.**
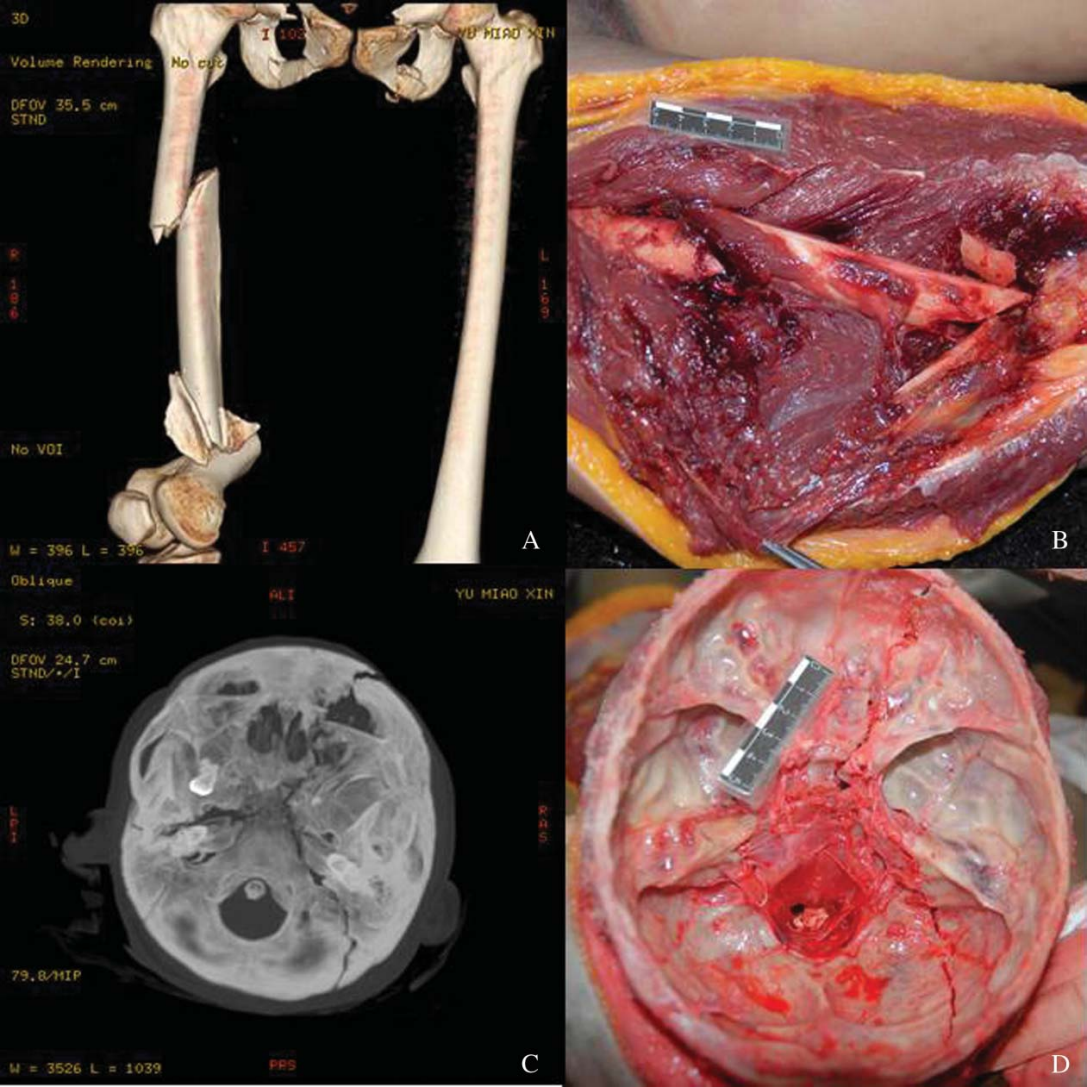
Comparison of PMCT and autopsy findings of victims in traffic accidents. (A and B) 3D reconstruction and autopsy showing comminuted fractures of the femur. (C and D) Maximum intensity projection images and autopsy showing fracture of the skull base.

Research into post-mortem forensic imaging started late in China, and the Institute of Forensic Science, Ministry of Justice, PRC (IFS), is the first organization systematically carrying out research. As the promoter and major research institution for post-mortem forensic imaging in China, IFS has commenced the studies on post-mortem forensic imaging, virtual human biomechanics and other digital technologies since 2005. Thereafter, as a key element of national natural science research, forensic post-mortem forensic imaging project has received instrumental, technical and financial support from hospitals, universities and government organizations. The research team conducted a comparative study between post-mortem forensic imaging and autopsy findings [12,51]. PMCT scans and autopsy were performed on the dozens of the bodies from the cases, in which victims died of mechanical injuries (including traffic accidents, falls from height, occupational accidents, intentional injury, etc.). Findings of the two methods were compared in aspects of the detection and reconstruction of injury and lesions. Results showed that most of the important information such as trauma, fracture and hemorrhage could be obtained by PMCT. Furthermore, PMCT can predict even minor injuries prior to autopsy, suggesting its practicability in the determination of the cause of death and injury manner. The comparative study between PMCT and autopsy on the bodies from several traffic accidents was carried out [5,52]. Results indicated that MDCT exhibits a relatively good detection on the details of fractures, tissue deformation and air accumulation in different body parts, is easy for analysis and data storage, and shows a high practical value in biomechanical accident reconstruction (Figure 1). MDCT and MRI images of hundreds of cases involving brain injury were retrospectively analysed, focusing on the characteristics of head injuries under different forces, injury-causing objects and injury manners [53]. Results suggested that forensic imaging approaches exhibit high sensitivity and accuracy in the diagnosis of head injuries and this technology can help the study on the biomechanical mechanism of head injury.

While IFS has been committed to the above researches, at the same time it gradually covered examination of drowned and burned bodies and comparison of injury-causing objects using PMCT, also conducting and developing new PMCTA approaches. Such approaches have been applied to actual cases, helped successfully determine the cause of death and injury manners, and provided scientific and objective forensic evidence [7,54–57]. Here are examples for using PMCT in cases of traffic accidents.

## Case examples 1–4

**Figure 2.**
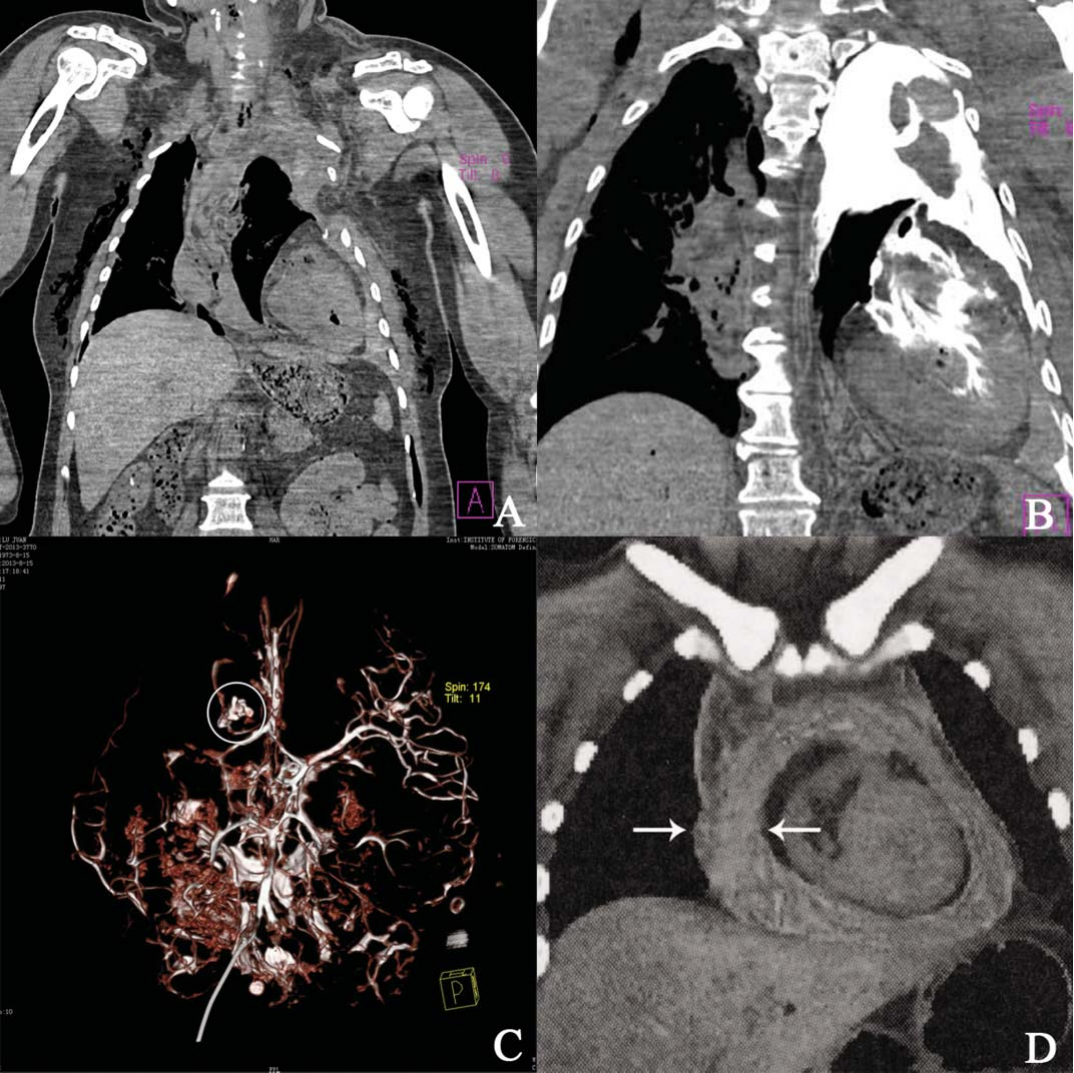
Application of PMCT and PMCTA in actual cases. (A and B) Detection of aortic rupture using PMCT and PMCTA by cardiac puncture. (A) PMCT revealed displacement of the heart into the left costophrenic angle. (B) Considerable leakage of contrast media into the left thorax was revealed. Only a small amount of contrast media entered the ascending aorta. (C) Diagnosis of a cerebral arteriovenous malformation using PMCTA of isolated brain. 3D reconstruction of PMCTA reveals the irregular and tangled vessels supplied from 1 branch of the right anterior cerebral artery (white circle). (D) Diagnosis of acute pericardial tamponade caused by blunt trauma to the chest. PMCT showing through pericardial effusion (arrows) causing compression of the heart.

**Figure 3.**
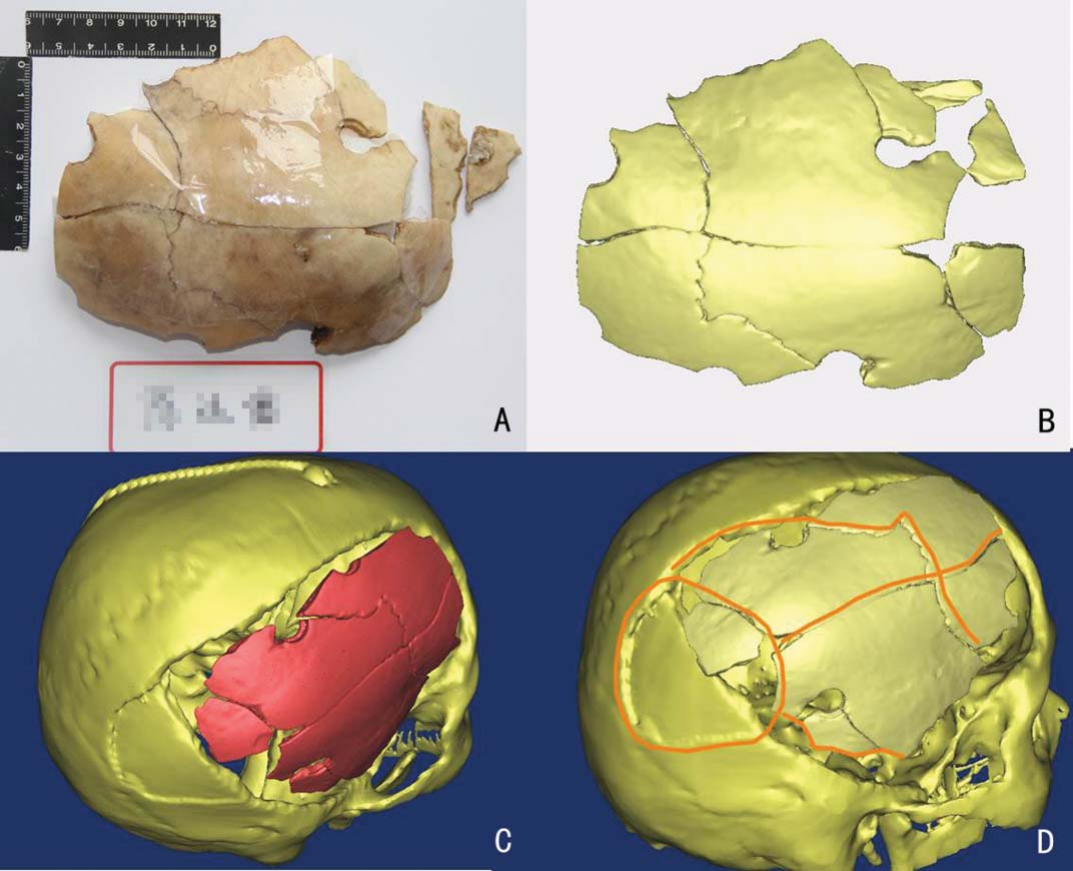
Application of MDCT and 3D reconstruction in the determination of injury manner. (A and B) The original and 3D reconstruction of the surgically removed skull segments using MDCT. (C) Restore of the complete skull by assembling the reconstructions of the bone fragment (red part) and the postoperative skull (yellow part). (D) Injury manner analysis according to the pattern and distribution of bone fractures (orange lines).

External examination followed by whole-body PMCT was performed on victims in four traffic accidents, two with additional PMCTA through cardiac puncture [58–60]. Rupture of the right ventricle and pericardial tamponade were found by PMCT and PMCTA in one case. The cause of death was related to the chest injury in the traffic accident. In another case, external examination of the body revealed scattered abrasions and contusions over the chest. Cutaneous emphysema of the chest wall, sternal fracture and bilateral clavicle and rib fractures, and hemopneumothorax as well as displacement of the heart into the left costophrenic angle were detected by PMCT. Then aortic rupture was confirmed by PMCTA. The cause of death was determined as hemorrhagic shock due to traumatic aortic rupture, probably being rolled over by wheels of the vehicle. Splenic rupture and peritoneal hemorrhage were found by MDCT in the third case. The cause of death was hemorrhagic shock resulted from abdominal injury in the traffic accident. In the fourth case, a 30-year-old male car driver died after an accident involving a collision with a guardrail barrier on a viaduct. A series of rib fractures and a transverse fracture of the sternum, pericardial tamponade and hemothroax were found by PMCT while external examination revealed only a minor skin abrasion in the middle of the sternum. The cause of death was considered as acute pericardial tamponade due to blunt chest trauma (Figure 2 and Figure 3). Autopsy of victims in traffic accidents was not enforced by the Chinese law and was rejected by the relatives in all the four cases. In certain circumstances, the combination of PMCT and PMCTA is of great helpful to the determination of the cause of death and injury manners in traffic accidents.

## Case example 5

PMCT was performed on a 45-year-old male driver who died in a traffic accident involving a collision of four cars [61]. No sign of injury was observed from the external examination and autopsy was rejected due to ethnic reasons. Dislocations of C_3-4_ and vertebrae fractures of C_4_, also spinal stenosis were detected during PMCT, suggesting severe whiplash injuries. PMCT was proved to be an effective and promising approach in the forensic investigation of cervical spine injury related to traffic accidents.

PMCT can also be used to better investigate injuries in order to obtain more information about the injury manner. Here are examples for using PMCT to determine injury manner.

## Case example 6

IFS utilized MDCT and 3D reconstruction to solve a craniocerebral trauma case in which a male sustained severe skull fractures and cerebral contusions during police interrogation [62]. Determination of whether the injury had been caused by a blow to the head or a fall was demanded. Because of the healing and craniocerebral surgery, the initial features of skull fractures were indistinguishable. Therefore, 3D reconstruction of the postoperative head and the surgically removed skull segments were made and assembled based on the MDCT data, revealing the original fractures and skull deformation. Based on the features of fractures observed in the restored skull model and cerebral contusions, the injury manner was considered to be a fall but not an intentional blow, thus resolving the conflicts between the victim's relatives and the police, also demonstrating the effectiveness of MDCT and 3D reconstruction in the forensic investigation of injury manner and exploration of injury mechanism.

## Case example 7

**Figure 4.**
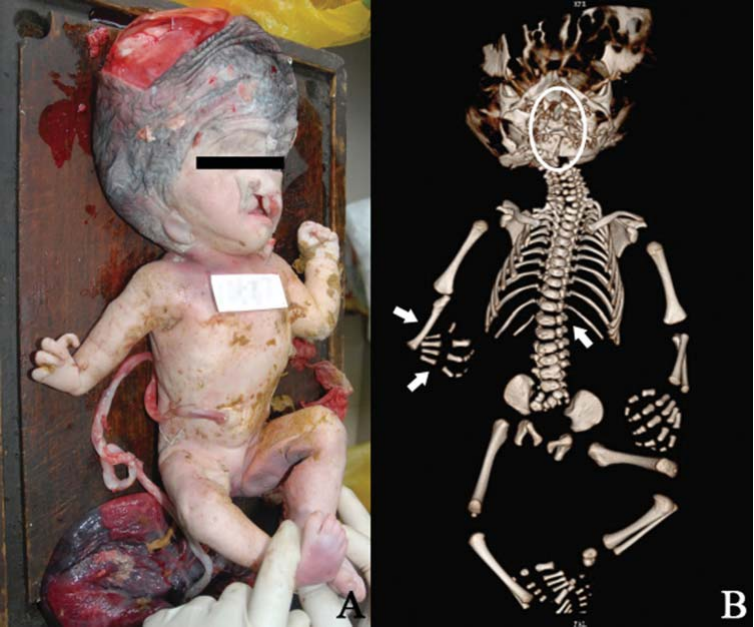
Determination of a newborn with lethal type IIosteogensis imperfect and other anomalies using PMCT and autopsy. (A) Anomalies in the head, face and extremities including a soft calvarium with fractures, a cleft lip, asymmetric ears, absence of a finger in right hand. (B) 3D reconstruction confirmed the fractured calvarium, a cleft palate and a cleft in the mandibular (circle), and absences of the left 12th rib, the right radial bone and first metacarpal bone (arrows).

PMCT followed by a medico-legal autopsy were performed on a stillbirth to explain the suspicious skull fractures and craniocerebral injuries during brutal delivery [63]. The autopsy findings showed typical features of lethal type II osteogenesis imperfecta (OI), including a soft calvarium, deformed extremities, flexed and abducted hips. PMCT findings included anomalies and variations as a cleft palate, mandibular dysplasia, spina bifida, costa cervicalis, and fusion of the ribs and vertebrae, which were difficult to detect during conventional autopsy (Figure 4). The results indicated that the death of the infant was unrelated to the medical practice, also showing great importance of PMCT to forensic examinations.

After a decade of research, IFS has performed and collected post-mortem body imaging data for hundreds of cases, including falls from height, traffic accidents, intentional injuries (mechanical injury and mechanical asphyxia), death caused by burning, drowning and electrocution, suspicion of medical malpractice and other types of cases. A body imaging database was preliminarily established. Currently, PMCT and PMCTA (if necessary) have become routine scans prior to autopsy in IFS, as well as an alternative when the cause of death cannot be determined during external examination of the body in traffic accidents and occupational accidents. Meanwhile, post-mortem forensic imaging has been recognized as a key research area in “Studies on the technologies to detect and identify the injuries in human body”– the National Key Technology R&D Program during the “twelfth five-year plan period”, and the standard practice for post-mortem forensic imaging was made and approved for implementation in November 2015 by Ministry of Justice, PRC.

In addition, post-mortem forensic imaging studies have been conducted in some research institutions of domestic public security bureaus and medical colleges for the judicial purpose of detecting injuries in the victim and for anatomy teaching [64–66].

## Finite element analysis (FEA) of human injury biomechanism based on post-mortem forensic imaging data

Post-mortem forensic imaging can only present morphology of injury, but not the dynamic deformation, displacement, strain and stress of human tissues when subjected to mechanical forces [8]. Such indicators can be calculated by FEA, carrying out in-depth study of the human injury biomechanics [67,68].

In recent years, medical imaging techniques, such as MDCT, MRI, have allowed to obtain data in DICOM format. By using commercial finite element modelling software, 3D finite element models of the human body can be established based on DICOM format images, with a high geometric and biomechanical fidelity [67,68]. By giving material properties, boundary conditions and external loads to the constructed finite element models, indicators like deformation, displacement, stress and strain of human tissues can be represented through computer simulation. Predictions of the injury location, morphology and severity can be delivered as reference for the forensic injury identification [69].

The FEA has been applied to the modelling and injury biomechanical study of human head, spine and other body parts since the 1970s [70–73]. Currently, the finite element modelling and analysis based on post-mortem forensic imaging data has played an important role in the study on injury biomechanics of different human organs and structures, and become a popular topic in the related research field [74–83].

Similar research started late in China. Since 2009, IFS has been the first and the only institution to apply FEA to forensic human injury biomechanical studies and actual cases. Based on PMCT data in DICOM format, IFS has established finite element models of the head, chest, pelvis, knee, lower limb and other parts and such models have been used to analysis the biomechanics of common mechanical injuries in forensic practice [84–87]. This approach helped to determine successfully the injury manners in several complicated and important cases in China, playing an important role in the forensic investigation. Two examples are given here.

## Case example 8

**Figure 5.**
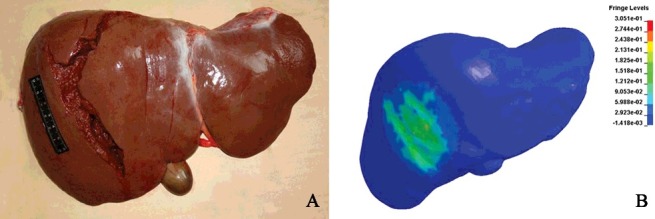
Finite element model of torso was used to simulate a lateral punch on the abdomen with an impact velocity of 6 m/s. Actual liver injury (A) was compared to the simulation results (B). The predicted region of greatest strain and location of injuries were indicated by high gradients of colour. Simulation results were highly consistent with the sites and severity of the actual liver injury.

A 30-year-old female died in the process of quarrelling with her boyfriend. Autopsy revealed a large area of liver rupture in the right lobe. During investigation, the police suspected that the victim sustained hand punches on the right hypochondrium by the boyfriend, but were confused about the possibility of a large area of liver rupture without being associated with rib fracture. IFS adopted a finite element model of torso to simulate scenarios involving hand punches on the front, right and rear sides of the abdomen with different velocities [88]. The simulation results showed that the punch on the right side was the most likely cause for liver damage, and a striking with a speed of above 6 m/s on the right side could lead to liver rupture at a large area and multiple sites, highly consistent with the actual liver injury of the victim (Figure 5). Simulation results also illustrated that since ribs have a high resistance to an impact, liver damage may not be associated with rib fractures.

## Case example 9

**Figure 6.**
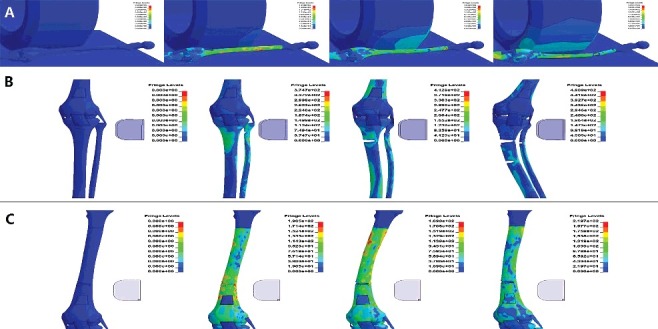
Finite element model of lower limb was used to simulate the scenarios involving the lower limbs being struck by bumper and rolled over by wheels. Von Mises stress distribution and injury patterns of lower limb at different time step in: (A) thigh under rolling over; (B) upper leg under direct impact; (C) lower thigh under direct impact. High gradients of colour indicated region of greatest stress and location of injuries were presented. Simulations of different scenarios resulted in different injury patterns.

IFS utilized a finite element model of lower limb to simulate the scenarios involving the lower limbs being struck by bumper and rolled over by wheels, determining the injury manner in an actual traffic accident [89]. The simulation results showed that when the lower limbs were rolled over by wheels, fracture occurred at the site of long bone contacting the edge of wheel, while the central section of the fractured bone remained relatively intact, thus resulting in segmental fractures. A typical wedge fracture would be formed under a side impact by the bumper, and it was difficult to form segmental fractures (Figure 6). The police was convinced by the comparison between the simulation results and actual injuries.

## Discussion

According to the foreign and Chinese research works, post-mortem forensic imaging can be used to exam the whole body, without loss or destruction of forensic evidence. It is repeatable, and can record the actual size of the lesions. The archived data can be used for consultation and re-examination, and 3D information can be provided as intuitive evidences in court [5,12,51,52]. Post-mortem forensic imaging can examine complicated body structures and areas not included in conventional autopsy, and can also be applied to ancient, highly decomposed bodies or bodies contaminated by infectious diseases, toxic substances, radionuclides, or other biohazards [2,9]. When there are strong cultural objections or objections from family members and it is not possible to perform autopsy, post-mortem forensic imaging can serve as an alternative method to provide a reference or even direct evidence for determining the cause of death and injury manners [8,10]. In some countries (not including China), findings from post-mortem forensic imaging and FEA have been recognized as legal evidence in court and can be used in the litigation and trials [10,90–93].

Post-mortem forensic imaging also has certain limitations, however. The quality of image acquired strongly depends on device performance, scanning parameters, body condition, the performance of the device-dependent software and operators’ personal judgments through the digital post-processing [60]. The image cannot present the true colour of tissues and artefacts may appear during the scanning. Currently, PMCT and PMMR cannot detect some small lesions and injuries that can only be found using a microscope [7,8,52,59]. In addition, post-mortem forensic imaging requires a costly equipment investment, and it is extremely difficult for general institutions to perform the related research.

FEA is also a virtual, non-invasive, repeatable approach. It has the ability to solve issues with complex structures, material properties and loading situations, showing apparent advantages in comparing different mechanisms of injury [69,94–97]. The existing finite element models of human body still need to be improved. Most of the existing models are patternized and factors are simplified during simulation. When applied in the analysis of the actual cases, the corresponding simulation results may maintain a certain deviation to the actual scenario [67,68]. However, the predictions of the force transmission path, and the concentrated area of stress and strain, injury area and morphology are accurate and reliable, which can be used as an important reference for the forensic determination of injury manner [98].

## Conclusion

As we have mentioned here, globally there have been lots of institutions carrying out research related to post-mortem forensic imaging and the followed FEA of injury. Numerous achievements have been made. Recently, similar research has been conducted by several institutions in China, making their own progress and contribution to the field. And like other technologies, post-mortem forensic imaging has both strengths and weaknesses.

The above-mentioned post-mortem forensic imaging and FEA are currently still in development stage in China. And the traditional forensic examination and research tools could not be completely replaced at present. However, the importance of those novel approaches will become increasingly prominent and recognized.
